# A longitudinal study of hippocampal subfield volumes and hippocampal glutamate levels in antipsychotic-naïve first episode psychosis patients

**DOI:** 10.1038/s41380-024-02812-1

**Published:** 2024-11-23

**Authors:** Eric A. Nelson, Nina V. Kraguljac, Adil Bashir, Stacey S. Cofield, Jose O. Maximo, William Armstrong, Adrienne C. Lahti

**Affiliations:** 1 Department of Psychiatry and Behavioral Neurobiology, University of Alabama at Birmingham, Birmingham, USA; 2 Department of Psychiatry and Behavioral Health, The Ohio State University, Columbus, USA; 3 Department of Biostatistics, University of Alabama at Birmingham, Birmingham, USA; 4 Department of Electrical and Computer Engineering, Auburn University, Auburn, USA

**Keywords:** Neuroscience, Schizophrenia

## Abstract

**Background:**

Previous studies have implicated hippocampal abnormalities in the neuropathology of psychosis spectrum disorders. Reduced hippocampal volume has been reported across all illness stages, and this atrophy has been hypothesized to be the result of glutamatergic excess. To test this hypothesis, we measured hippocampal subfield volumes and hippocampal glutamate levels in antipsychotic naïve first episode psychosis patients (FEP) and the progression of volume decline and changes in glutamate levels over a 16-week antipsychotic drug (APD) trial. We aimed to determine if subfield volumes at baseline were associated with glutamate levels, and if baseline glutamate levels were predictive of change in subfield volumes over time.

**Methods:**

We enrolled ninety-three medication-naïve FEP participants and 80 matched healthy controls (HC). T1 and T2 weighted images and magnetic resonance spectroscopy (MRS) data from a voxel prescribed in the left hippocampus were collected from participants at baseline and after 6 and 16 weeks of APD treatment. Hippocampal subfield volumes were assessed using FreeSurfer 7.1.1., while glutamate levels were quantified using jMRUI version 6.0. Data were analyzed using linear mixed models.

**Results:**

We found regional subfield volume deficits in the CA1, and presubiculum in FEP at baseline, that further expanded to include the molecular and granule cell layer of the dentate gyrus (GC/ML/DG) and CA4 by week 16. Baseline hippocampal glutamate levels in FEP were not significantly different than those of HC, and there was no effect of treatment on glutamate. Glutamate levels were not related to initial subfield volumes or volume changes over 16 weeks.

**Conclusion:**

We report a progressive loss of hippocampal subfield volumes over a period of 16 weeks after initiation of treatment, suggestive of early progression in neuropathology. Our results do not suggest a role for glutamate as a driving factor. This study underscores the need to further research the mechanism(s) underlying this phenomenon as it has implications for early intervention to preserve cognitive decline in FEP participants.

## Introduction

Previous studies have implicated hippocampal abnormalities in the neuropathology of psychosis spectrum disorders [1–4]. Reduced hippocampal volume has been reported across illness stages, including in high-risk subjects, first episode psychosis (FEP), and chronic psychosis [1, 5–17]. At a more granular level, hippocampal subfields [18, 19] detected volume deficits throughout multiple hippocampal subfields across illness stages [7, 12, 20–24], including the hippocampal tail, CA1, CA2/3, CA4, molecular layer, dentate gyrus (DG), subiculum, and presubiculum [7, 12, 20–26]. Further evidence suggests volume deficits are initially localized to the CA1 region early on but subsequently spread to other subfields as the illness progresses [17, 26–31]. Not all studies have replicated findings of CA1 deficits. One study found deficits in the CA2/3, CA4/DG with no differences in CA1 [28], though FEP patients did show CA1 volume reduction compared to chronic patients [28]. Another study found only increased CA3 volume in FEP patients [32]. These findings are critically important as reduced hippocampal volume is associated with poor memory performance across psychosis stages [9, 33–36]. As for subfields, reduced CA4/DG volume was shown to be associated with poor visuospatial memory in chronic and recent onset psychosis patients compared to controls [28], while poor visual learning was linked independently to reduced CA2/3 and CA4/DG volume in both schizophrenia and ultra high-risk (UHR) groups [25]. Understanding the mechanisms underlying these deficits might help with the identification of specific therapeutic targets that could alleviate or even arrest structural disease progression.

The hippocampus is particularly susceptible to glutamatergic imbalance due to its densely packed glutamatergic pyramidal neurons [37–39] regulated by GABAergic interneurons [38, 39]. A central theory in schizophrenia postulates excitation/inhibition imbalance as a potential causative factor, where glutamate (Glu) excess could result in atrophy [17]. Consistent with this idea, our data in chronic unmedicated patients showed that Glu was negatively correlated with whole hippocampus volumes [20]. In this context, it is important to note that studies investigating hippocampal Glu using magnetic resonance spectroscopy (MRS) have been rare, and findings are mixed [2, 4, 7, 20, 40–43]. In parallel, longitudinal studies have been minimal, finding that elevated glutmate + glutamaine (Glx) levels at baseline were not affected by a six-week course of antipsychotic medication [4]. In this study we used magnetic resonance spectroscopy (MRS) to investigate hippocampal glutamate levels though other methods exist that can interrogate the glutamate [44] and the GABA [45] systems.

Together these data suggest that glutamatergic pathology and atrophy patterns may evolve over time and/or be sensitive to antipsychotic medication exposure, but due to the sparsity of longitudinal studies, this remains poorly understood.

In this longitudinal study of medication-naïve FEP and matched healthy controls (HC) we measured hippocampal subfield volumes and hippocampal Glu levels at three time points; prior to antipsychotic drug (APD) treatment in FEP as well as at 6 and 16 weeks after treatment with risperidone. The goals of this project were threefold. First, we sought to determine the baseline hippocampal subfield volumes to test the hypothesis that patients would show localized deficits in the CA1 [17, 26–31] and elevated Glu levels [17, 39, 46, 47]. Second, we aimed to characterize the early progression of subfield volume decline, measured over at least two time points, and changes in Glu levels in FEP patients over the 16-week trial. We hypothesized that hippocampal volume deficits would become more extensive and spread to additional subfield regions, and that Glu levels would remain unchanged [4]. Third, we sought to determine the relationship between these two measures at baseline and determine if baseline Glu levels would predict changes in subfield volumes over time. We hypothesized that there would be a negative association between baseline hippocampal and subfield volumes and Glu levels, and that higher baseline Glu would be predictive of a greater decrease in hippocampal and subfield volumes.

## Methods

### Participants

Ninety-three antipsychotic-naive FEP were recruited from the emergency room, inpatient units, and outpatient psychiatry clinics at the University of Alabama at Birmingham (UAB) in this UAB Institutional Review Board approved study. Written informed consent was obtained prior to enrollment and after participants were deemed to have capacity to provide consent [48]. Consensus diagnoses were made according to DSM-5 criteria by two board certified psychiatrists (ACL and NVK) taking into consideration information from the Diagnostic Interview for Genetic Studies (DIGS) or Mini-International Neuropsychiatric Interview (MINI) and medical records as available. In addition, because of the longitudinal design of the study (ClinicalTrials.gov Identifier: NCT02034253, NCT03442101) clinical observations over several months of follow up were used to establish a final diagnosis. The Brief Psychiatric Rating Scale (BPRS) was used to assess symptom severity [49]. Cognitive function was assessed using the Repeatable Battery for the Assessment of Neuropsychological Status (RBANS) [50] at the beginning of the study. Duration of untreated Psychosis (DUP) was also assessed and defined as the time (in months) between the first onset of discernable positive symptoms to the time of initial treatment contact, which also coincided with enrollment in the study. Exclusion criteria included major neurological or medical conditions, a history of head trauma with loss of consciousness, substance use disorders (excluding nicotine among FEP and HC, and cannabis use among FEP), within one month of imaging, pregnancy or breastfeeding, or MRI contraindications.

FEP were enrolled in a 16-week open-label, risperidone trial using a flexible dosing regimen. Risperidone started at 1 to 3 mg and titrated in 1 to 2 mg increments; pill counts were done to monitor compliance. Three FEP participants switched from risperidone to aripiprazole prior to 16 weeks. Use of concomitant psychotropic medications was permitted as clinically indicated. The number of FEP participants taking concomitant medications at any time during this study included: amitriptyline (1), amphetamine salts (1), benztropine (23), bupropion (3), buspirone (1), diphenhydramine (2), hydroxyzine (2), lithium (1), lorazepam (7), SSRIs (32), prazosin (2), propranolol (3), trazodone (9), and valproate (1).

Additionally, 80 HC matched on age, sex, and parental socioeconomic status (SES) were also recruited by advertisements (flyers). In addition to the above-outlined criteria, HC with a personal history or a family history of a psychosis spectrum disorder in a first-degree relative were also excluded. Likewise, they were scanned three times over a 16-week period. Data for both groups were collected roughly in tandem from April 2015 to May 2021. HCs were scanned as soon a suitable match for each FEP participant could be recruited.

### Data acquisition and preprocessing

#### Structural MRI

Participants were scanned on a whole-body 3T Siemens MAGNETOM Prisma MRI scanner using a 20-channel head coil. Anatomical scans were acquired via T1-weighted (TR/TE = 2400/2.22 ms, flip angle 8°, 0.8 mm isotropic voxels) and T2-weighted images (TR/TE = 3200/563.0 ms, flip angle 8°; 0.8 mm isotropic voxels). To improve the accuracy of hippocampal segmentation [51] participants’ T1- and T2-weighted images were used during preprocessing with FreeSurfer 7.1.1 [52]. Freesurfer’s longitudinal hippocampal subfield segmentation does not support T2-weighted images. As a result, our data was preprocessed cross-sectionally. We did not use FreeSurfer’s skullstripping as we found a handful of patients did not skull strip properly. Instead, we skullstripped all structural scans using FSL 6.0.3 prior to FreeSurfer processing. We used FreeSurfer’s hippocampus subfield segmentation module to calculate each participant’s left and right subfield volumes [51]. Bilateral subfield volumes were then assessed by additively combining these volumes. Estimated total intracranial volume (ICV) [53] was also obtained. Quality control was assessed via the ENIGMA structural image processing protocol (http://enigma.usc.edu/) [54]. We quantified overall hippocampal volumes, and volume of eight hippocampal subregions that have been found to be altered in patients with psychosis [23, 25, 26]: the hippocampal tail, CA1, CA3 (the combined CA2/CA3 region), CA4, the molecular layer (ML), the molecular and granule cell layer of the DG (GC/ML/DG), subiculum, and presubiculum. Due to concerns regarding the reliability of segmentation results from smaller subfields [25, 55] the fimbria, hippocampal fissure, and parasubiculum were not included in our analysis.

#### Proton magnetic resonance spectroscopy (MRS) acquisition

Data were collected from a voxel in the left hippocampus (27 × 15 × 10 mm^3^; Fig. S1). A combination of automatic and manual shimming was used to minimize the linewidth. Spectra were then obtained using a point-resolved spectroscopy sequence (PRESS TR/TE = 2000/80 ms, flip angle = 90°, 192 averages; TE was chosen in order to resolve and separate the C4 resonance of Glu from other jcoupling metabolites [56, 57]). Water suppression was achieved by WET approach using CHESS pulses. Eight averages of unsuppressed water were acquired as a reference.

#### MRS preprocessing

All spectra were preprocessed in jMRUI version 6.0 using the QUEST algorithm [58, 59]. The basis set was simulated using the timing parameters of the PRESS sequence. The simulation consisted of peaks for N-acetyl aspartate, choline, creatine, and Glu. Glutamine was also included in our basis set as a metabolite of no interest, separate from Glu to increase the quality of the Glu signal. The position (frequency) and linewidths of individual metabolites were independently adjusted to fit the data and the Subtract approach was used for background handling. After removing the residual water peak using the Hankel-Lanczos singular values decomposition filter, the amplitude for Glu was estimated and then calculated relative to the unsuppressed voxel water and expressed in institutional units [60]. Voxel tissues were segmented using the Gannet toolbox (version 3.1) [61]. Glu levels were then corrected for partial volume effects according to Gasparovic and colleagues [62, 63]. Exclusion criteria for Glu failure in fitting the QUEST algorithm included signal to noise ratio <3, full width at half maximum (FWHM) > 0.1 ppm [64], and Cramer–Rao lower bounds (CRLB) > 20%.

### Statistical analyses

**Fig. 1:**
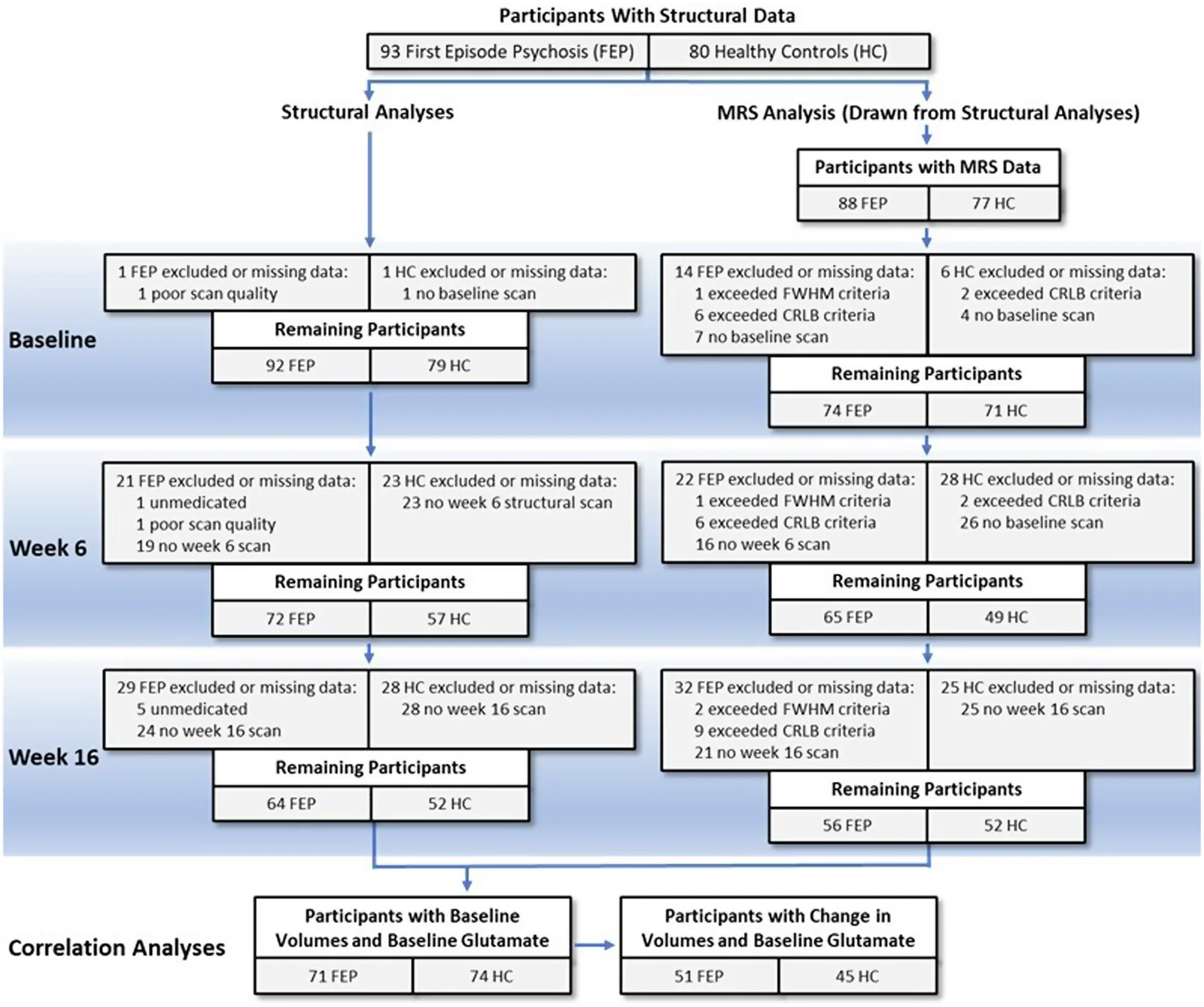
Consort flow chart. Presents the number of participants in each of our 10 mixed models (Total hippocampal volume, 8 hippocampal subfield volumes and hippocampal glutamate) at each time point. The flow chart also shows the number of excluded participants in each model at each timepoint and reasons for exclusion. Lastly the flow chart shows the number of participants for our glutamate × volume correlation analyses.

Figure 1 provides a consort flow chart for our primary analyses, additionally a full description of the number of participants per analysis as well as the number and reasons for any participant exclusions from any analysis are described in the supplemental section. General linear mixed models and regression analyses were performed using JMP 17 pro (Cary, NC). All other analyses (bivariate and partial correlations, *t*-tests and chi-square tests) were performed using SPSS 29. *P*-values < 0.05 were considered meaningful.

#### Baseline hippocampal volumes and glutamate, and change over time

Generalized linear, repeated measures mixed models, with AR(1) covariance structure, repeating on participant, were used to determine if hippocampal and subfield volumes, as well as hippocampal Glu levels, differed between FEP and HC groups over time. Fixed effects included group (HC and FEP), time (baseline, week 6, and week 16), group x time, and covariates. Covariates for volume models included age [65] and ICV. Sex showed high correlation with ICV but accounted for less volume variance and was therefore not included. Covariates for our Glu model included sex [66], smoking status [67, 68], and FWHM (based on an observed baseline group difference). Age and sex were correlated, and age accounted for less glutamate variance and was therefore not included. Due to high correlation between volume measures (all *p* < 0.0001, data not shown) model fixed-effect F-tests were not corrected across models. Within models, when warranted, multiple comparisons were corrected using Tukey’s HSD. No imputation for missing data was used.

#### Completers and subgroup analyses

The above mixed models were also repeated with similar covariates and effects, as a completers analysis (only participants with all data points were included). This reduced the participant N to 31 HC and 35 FEP. The above mixed models were also repeated excluding HC and splitting FEP into two subgroups: schizophrenia FEP (SZ-FEP, *N* = 48) and non-schizophrenia FEP (NSZ-FEP, *N* = 45).

#### Relationship between hippocampal volumes and glutamate

Covariate adjusted linear regression was used to assess if baseline hippocampal and subfield volumes were related to baseline hippocampal Glu levels, and if baseline Glu levels were predictive of change in hippocampal and subfield volumes at week 16 (*Week* 16 – *Baseline*). HC and FEP were analyzed separately. Covariates included smoking status and sex. ICV and age showed collinearity issues with sex and were not included. FWHM was not included since models were assessed by HC and FEP separately.

#### Exploratory analyses

Partial correlation analyses was used to examine the relationship between Glu and BPRS scores (total as well as positive and negative subscales) at baseline and week 16 respectively and also assessed whether baseline Glu was a predictor of treatment response (based on % change in BPRS positive score from [A] baseline to [B] week 16: (((B‐A)/A)*–100)), controlling for smoking and gender. Similarly, partial correlations were used to examine the relationship between subfield volumes and BPRS and RBANS scores at baseline and week 16 respectively and assessed whether any subfield volumes were predictive of treatment response, controlling for age and ICV (at baseline and week 16 respectively). Partial correlation analysis was also used to assess the relationship between DUP and baseline subfield volumes and to assess whether DUP was predictive of subfield volumes at week 16. Age and ICV (baseline or Week 16 respectively) were entered as covariates.

Bivariate correlations were used to investigate the relationships between several clinical measures including RBANS (total and subscales) with DUP, RBANS (total and subscales) with baseline and week 16 BPRS (total and subscales), RBANS (total and subscales) with treatment response, and DUP with treatment response.

Two-tailed independent sample *t*-tests were used in FEP to assess if there were any differences between left and right subfield volumes at baseline, and for lateralized differences between baseline anterior and posterior hippocampal volumes in FEP.

## Results

### Demographics

No significant group differences were observed for sex, age, parental socioeconomic status at baseline (Table 1) or week 16 (Table S21). FEP smoked more packs per day compared to HC. As expected, HCs scored higher on the RBANS than FEP.

**Table 1:** Demographics, clinical measures, and MRS signal criteria.

	FEP (*n* = 93)	HC (*n* = 80)	*t*/*χ*^*2*^	*p*
Sex (%male)	65.59	58.75	0.858	0.354
Age	23.35 (5.79)	24.21 (5.53)	0.992	0.322
Race (Caucasian/African American/Other)	34/53/6	43/18/19	24.255	<0.001
Parental SES^a^	5.41 (4.47)	4.50 (3.87)	23.576	0.099
Packs per day^b^	0.24 (0.47)	0.02 (0.07)	4.567	<0.001
Diagnosis				
Schizophrenia	48			
Schizoaffective disorder	17			
Schizophreniform disorder	4			
Brief psychotic disorder	3			
Bipolar disorder with psychosis	3			
Major depression with psychosis	3			
Psychosis not otherwise specified	15			
DUP [in months; range, median, mean, (SD)]	0.25–180; 7.00; 25.15; (42.14)			
Risperidone dosage^c^				
Week 6	4.10 mg			
Week 16	4.47 mg			
UDS + cannabis (%)^d^	37.30			
BPRS^e^				
Total				
Baseline	48.00 (11.86)			
Week 6	32.54 (8.50)			
Week 16	29.33 (5.92)			
Positive				
Baseline	11.00 (3.38)			
Week 6	5.43 (2.95)			
Week 16	4.53 (2.04)			
Negative				
Baseline	5.48 (3.09)			
Week 6	5.55 (2.67)			
Week 16	5.44 (2.69)			
RBANS^f^				
Total	74.43 (15.16)	92.74 (10.81)	8.638	<0.001
Immediate memory	82.66 (17.57)	101.55 (15.94)	6.864	<0.001
Visuo-spatial	74.13 (16.95)	82.88 (13.88)	3.429	<0.001
Language	84.90 (16.82)	96.81 (15.33)	4.511	<0.001
Attention	80.06 (17.57)	102.46 (15.61)	8.211	<0.001
Delayed memory	77.10 (15.29)	90.71 (9.51)	6.672	<0.001
SNR^g^				
Baseline	11.75 (1.93)	11.92 (1.86)	0.534	0.594
Week 6	12.32 (1.28)	11.96 (1.05)	–1.589	0.115
Week 16	12.34 (1.18)	12.28 (1.10)	–0.295	0.385
FWHM (Hz)^g^				
Baseline	5.26 (2.15)	4.81 (1.87)	–1.354	0.089
Week 6	4.81 (1.20)	4.58 (1.24)	–0.987	0.326
Week 16	4.75 (0.95)	4.63 (1.09)	–0.637	0.526
Glutamate CRLB^g^				
Baseline	12.58 (2.94)	11.52 (2.69)	–2.269	0.025
Week 6	12.39 (3.07)	12.38 (3.05)	–0.029	0.977
Week 16	12.67 (2.71)	11.77 (2.78)	–1.695	0.093

Values are mean (SD) or %.

*BPRS* brief psychiatric rating scale (positive subscale included conceptual disorganization, hallucinatory behavior, and unusual thought content; negative subscale included emotional withdrawal, motor retardation, and blunted affect), *CRLB* Cramér–Rao lower bound, *DUP* duration of untreated psychosis, *FEP* first-episode psychosis, *HC* healthy control subjects, *MRS* magnetic resonance spectroscopy, *RBANS* repeatable battery for the assessment of neuropsychological status, *SES* parental socioeconomic status, *SNR* signal to noise ratio, *UDS* urine drug screen.

^a^SES determined from Diagnostic Interview for Genetic Studies (1–18 scale); lower numerical value correspond to higher socioeconomic status; 8 FEP lacked SES scores, *N* = 85; 4 HC lacked SES scores, *N* = 76.

^b^1 FEP lacked smoking data, *N* = 92.

^c^Week 6 *N* = 67; Week 16 *N* = 55.

^d^10 FEP lacked cannabis data.

^e^17 FEP lacked BPRS scores at week 6, *N* = 76; 23 FEP lacked BPRS scores at week 16, *N* = 70.

^f^11 FEP lacked RBANS scores, *N* = 82; 11 HC lacked RBANS scores, *N* = 69.

^g^FEP baseline *N* = 74, week 6 *N* = 66, and week 16 *N* = 56; for HC baseline *N* = 71, week 6 *N* = 49, and week 16 *N* = 52.

### Longitudinal analyses

**Fig. 2:**
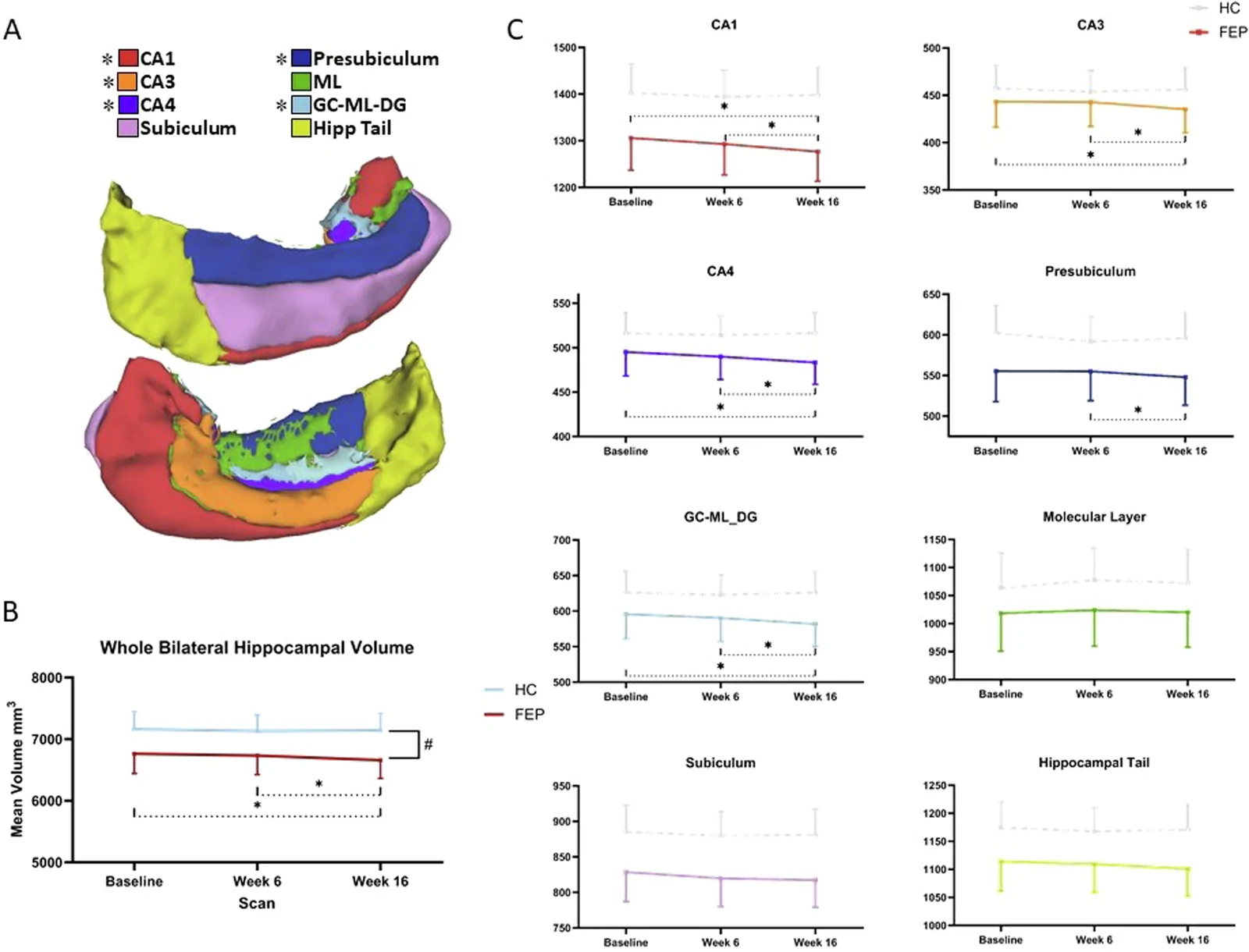
Hippocampal subfield volume changes over time in first episode psychosis. **A** shows a 3D model of the segmentation of all hippocampal subfields segmented for this study color coded by region. Colored boxes marked by an * signify regions with significant group × time interactions (*p* < 0.05 after Tukey’s HSD). **B** A scan time × volume graph of the whole bilateral hippocampal volume over time comparing FEP to HC. Post hoc tests showed a significant difference in the main effect for group (*p* < 0.05 after Tukey’s HSD) with FEP showing greater overall reduction in whole hippocampal volume compared to HC. Meanwhile * signifies a significant difference between baseline and week 16, and week 6 and week 16 within FEP (*p* < 0.05 after Tukey’s HSD). **C** Within FEP volume differences over time for each subfield. In short FEP showed a consistent reduction in volume over time for the whole hippocampus as well as the CA1, CA3, CA4, presubiculum, and GC-ML-DG hippocampal subfields.

Results from group × time general linear mixed models for volume showed significant group × time interactions for the whole hippocampus as well as the CA1, CA3, CA4, presubiclum, and GC/ML/DG subfields (*p* < 0.05; Table S1, Fig. 2A). Results from the group × time general linear mixed model for Glutamate also showed a significant interaction (*p* < 0.05 Table S3).

#### Baseline group comparisons

**Fig. 3:**
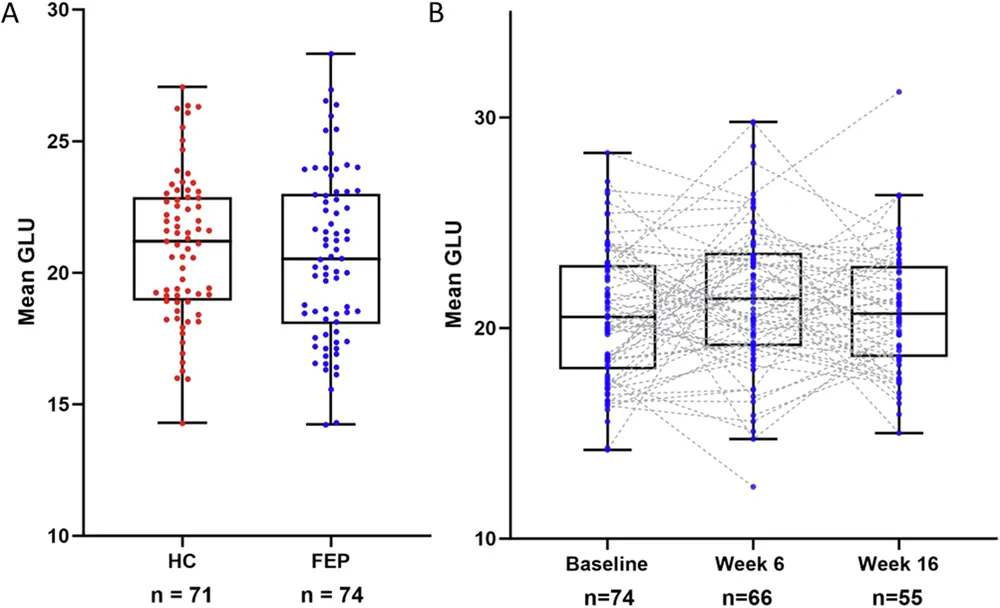
Baseline hippocampal glutamate group difference and change in FEP glutamate over time. Hippocampal glutamate did show significant group × time interaction, *F*(2,46.9) = 4.60 *p* = 0.0109. **A** Glutamate comparisons between HC and FEP showed no significant group differences at baseline. **B** FEP demonstrated no significant change in glutamate level over time in pot-hoc analyses.

We hypothesized that patients would show localized deficits in the CA1 and elevated Glu levels. Our within model post-hoc comparisons for volume showed that at baseline FEP had significantly reduced volume in the CA1 and presubiculum (Table S2, Fig. 2C). Within model post-hoc comparisons did not show a significant group difference in Glu at baseline (Table S4, Fig. 3A) or any other time point (Table S4).

#### Change over time

We hypothesized that hippocampal volume deficits would become more extensive and spread to additional subfield regions, and that Glu levels would remain unchanged. Post-hoc comparisons showed that in FEP CA1 and presubiculum deficits still persisted while deficits were also seen in the CA4 and the GC/ML/DG at week 16 (Fig. 2C, Table S2). These post-hoc comparisons further showed a consistent reduction in all volumes except the presubiculum from baseline to week 16 and all volumes from week 6 to week 16. (Fig. 2C, Table S2). Within model post-hoc comparisons for Glu did not show significant change in Glu in FEP over time (Table S4, Fig. 3B).

### Relationship between subfield volumes and glutamate

Lastly, we hypothesized that there would be a negative association between baseline hippocampal and subfield volumes and Glu levels, and that higher baseline Glu would be predictive of a greater decrease in hippocampal and subfield volumes. Unadjusted *p*-values showed significant correlations between baseline presubiculum (*p* = 0.0173) and total hippocampus (*p* = 0.0449) values and baseline Glu, but this significance did not persist after adjustment for covariates (*p* = 0.6296, and *p* = 0.9301, respectively), specifically after adjusting for sex (Table 2). Baseline Glu levels did not predict changes in hippocampal or subfield volumes after 16 weeks (Table 2). Unadjusted correlations of baseline Glu with baseline volumes and change in volumes in HC were not significant (Table S5). Because of this there was no need to adjust for covariates.

**Table 2:** Glutamate and volume correlations in FEP.

Baseline glutamate × baseline volume						
	CA1	CA3	CA4	Presub	GC/ML/DG	Total Hipp
*r*	0.2130	0.1494	0.1414	0.2571	0.1477	0.2231
95%CI	–0.0039 0.4311	–0.1142 0.3458	–0.0796 0.3674	0.0506 0.4745	–0.0722 0.3739	0.0056 0.4389
unc. *p*	0.0542	0.1773	0.1970	**0.0173**	0.1761	**0.0449**
sex *p*	**<0.0001**	**<0.0001**	**<0.0001**	**0.0011**	**<0.0001**	**<0.0001**
PPD *p*	0.3256	0.1021	0.0018	0.5161	0.0017	0.0292
Glu *p*	0.5347	0.4595	0.6343	0.6296	0.6407	0.9301
**Baseline glutamate** **×** **change in volume (Week 16 – Baseline)**						
*r*	–0.1441	0.0232	–0.1104	0.1046	–0.1129	–0.0954
95%CI	–0.4036 0.1369	–0.2540 0.2969	–0.3746 0.1704	–0.1760 0.3695	–0.3767 0.1679	–0.3615 0.1850
unc. *p*	0.3131	0.8714	0.4406	0.4651	0.4304	0.5054
sex *p*	0.7407	0.1998	0.8717	0.3334	0.4206	0.3029
PPD *p*	0.7795	0.3393	0.8436	**0.0272**	0.7641	0.3539
Glu *p*	0.2474	0.4373	0.4239	0.4546	0.9138	0.1792

The unc. *p* shows glut × volume *p* values before adjusting for covariates. Glu *p* shows glutamate × volume *p* values after adjusting for covariates. 95% confidence intervals, low (top) to high (bottom) range. Bold values indicate significance, *p* < 0.05.

### Additional results

#### Partial correlations

Glutamate was not associated with BPRS at either timepoint, nor was baseline glutamate predictive of treatment response (Table S6). Greater GC-ML-DG (*p* = 0.035) and hippocampal tail (*p* = 0.043) volumes at week 16 were associated with greater negative BPRS scores at week 16 and greater baseline GC-ML-DG (*p* = 0.029) volume was predictive of better response to treatment (Table S7). In FEP, RBANS visuospatial scale showed a significant correlation with baseline molecular layer volume (*p* = 0.043), while the RBANS immediate memory subscale was significantly correlated with week 16 CA3 (*p* = 0.046) and molecular layer (*p* = 0.009) volumes (Table S8A). In HC, RBANS immediate memory scale was correlated with baseline CA3 (*p* = 0.034) volume (Table S8B).

#### Bivariate correlations

DUP was not related to RBANS (total or subscales) (Table S9). Higher RBANS immediate memory (*p* = 0.049) and delayed memory (*p* = 0.028) scores were predictive of lower total BPRS score at week 16, while higher delayed memory (*p* = 0.005) was also predictive of lower BPRS positive score at week 16. However, RBANS and its subscales were not associated with baseline BPRS scales or treatment response (Table S10). Shorter DUP was predictive of better treatment response (*r*(68) = 0.255, *p* = 0.033), a result widely replicated in the literature [69, 70]. Longer DUP was associated with reduced baseline volumes of the subiculum (*p* = 0.039) and hippocampal tail (*p* = 0.046). DUP was not predictive of subfield volumes at week 16 (Table S11).

#### Completers, subgroup and lateralization analyses

In the completers analysis significant group × time interactions for CA1 and presubiculum volumes did not persist, though there was a significant main effect for group in the presubiculum model (Table S12). Significant group × time interactions for CA3, CA4, and GC/ML/DG volumes did remain (Table S12), but post hoc tests showed no significant between group or within group comparisons for these regions (Table S13). The presubiculum did show significant group difference in volume at baseline and week 16 (Table S13). Results from the completers Glu mixed model showed a significant group x time interaction again (Table S14), but all post hoc comparisons were not statistically significant except for a difference in HC Glu from week 6 to Week 16 (Table S15).

For Subgroup models (SZ-FEP vs NSZ-FEP), a significant group difference was seen in the subiculum and molecular layer, but post hoc tests showed no significant group comparisons. Significant main effects for time were found in the CA1, CA4, subiculum, presubiculum, and GC/ML/DG models (Table S16). Further, SZ-FEP showed significant changes in CA1, CA4, and GC/ML/DG models between baseline and week 16. NSZ-FEP only showed a significant change in total hippocampal volume between week 6 and week 16 (Table S17). The subgroup model for Glu showed no significant interaction of main effects (Table S18).

Results from Table S19 showed that the right CA1 and CA3 and the left presubiculum were significantly larger in FEP and left posterior and anterior hippocampal volumes were greater than the right in FEP (Table S20).

## Discussion

To our knowledge, this is the first multimodal, longitudinal study investigating the early time course of hippocampal subfield volumes and Glu pathology in a large group of medication naïve FEP. Consistent with our hypotheses, we found that volume deficits in the CA1 and presubiculum were already present at baseline, and that atrophy spread to the GC/ML/DG and CA4 by week 16. In contrast, we found no hippocampal Glu alterations in patients at baseline or change in levels after sixteen weeks of treatment. We also did not note any relationships between hippocampus subfield atrophy and Glu levels, or cognitive deficits.

For the completers analyses, while direction of association remained, due to drastically reduced sample size, most statistical associations did not persist. Subgroup models (SZ-FEP vs NSZ-FEP) also provided few significant results.

### Hippocampus subfield atrophy

As hypothesized, FEP had baseline volume deficits in the CA1 and the presubiculum. Cerebral blood volume (CBV) abnormalities selective to the CA1 subfield appear to exist prior to the development of psychosis [17]. Exhibited by an animal model using an NMDA antagonist to test if glutamate elevation acts as a driver for those abnormalities reproduces a similar regional pattern of metabolic abnormalities [17]. It was suggested [26] that greater density of glutamate receptors in the CA1 region compared to other subfields could make this region more vulnerable to glutamate-induced neurotoxicity [71]. In addition, selective decline in CA1 subfield volume is also seen in individuals at ultra-high-risk (UHR) for psychosis whose symptoms persist and in those who develop psychosis [72]. Cross-sectional studies show volume deficits selective to the CA1 subfield in the early stage of psychosis and progression to other subfields in chronic psychosis [26]. Our results reflect these findings and fit well with other data showing that early CA1 deficits seen in the prodromal and attenuated psychosis stages spreads to the subiculum as the illness progresses to syndromal psychosis [17, 31]. Similar to many [30, 73–76], but not all [27, 34, 77, 78] studies evaluating the evolution of structural hippocampus pathology, we show a progressive decline in hippocampal subfield volumes over time. The spreading decline in volume to additional subfields is consistent with another report of subfield volume deficits in a community sample of FEP who had been treated for less than three months at the time of the assessment [79] and a longitudinal investigation demonstrating early CA1 deficit followed by progressive atrophy across subfields [26]. Of course, it is difficult to disentangle the natural progression of the illness from direct effects of APD, as it would be unethical to withhold treatment from patients. Studies on this topic thus far are inconsistent [12, 24, 80], with reports of a negative [11, 13, 73, 76, 81] or positive relationship [80, 82] between hippocampus volume and treatment, and it remains unclear if APD treatment preferentially impacts some subfields, but not others [83].

Our FEP lateralization results did not show the same widespread left hemisphere volume deficits expected based on a recent meta-analysis [1]. Though unexpected these results are not novel. Another recent study comparing treated and untreated schizophrenia patients showed greater left hippocampal and subfield volume in never treated schizophrenia patients while patients who had been treated had greater left hippocampal subfield volumes [12]. Such results along with our own point to the significant heterogeneity seen within the psychosis population.

### Hippocampus glutamate

Based on our prior studies in unmedicated schizophrenia patients and a ketamine model, both finding an increase in hippocampus Glx and suggestive of NMDA receptor hypofunction as a key pathophysiological mechanism [20, 84], we hypothesized that Glu levels would be elevated in our current sample. While we did not report baseline hippocampus Glu abnormalities here, we speculate that it is possible that either Glu levels need to be placed in the context of GABA levels to fully capture an early disruption in the excitation/inhibition balance, or that the spatial resolution of the spectroscopy voxel measuring Glu in the whole hippocampus was insufficient to detect Glu pathology that is only present in one or a few subfields.

To our knowledge, this is the largest cohort of medication naïve FEP used to date to evaluate hippocampal Glu, and we found no effects of APD treatment. This is consistent with one of our prior studies in a non-overlapping sample of schizophrenia spectrum disorder patients that found no change in Glu levels after six weeks of treatment with risperidone [4]. An earlier longitudinal study on effects of APD in FEP reported normalization of thalamic Glu [85], as well as anterior cingulate cortex Glu levels lower at baseline and follow up [85]. Collectively these data suggest baseline and longitudinal changes in Glu in FEP are region-specific.

### Relationship between hippocampus subfield volumes and glutamate

Here, we found no relationship between subfield volumes and Glu in patients, nor was Glu a predictor of further volume decline, which contrasts our previous study in unmedicated patients where Glx levels were increased and negatively correlated with whole hippocampal volume [20]. Again, it may well be that it is the excitation/ inhibition imbalance that dives atrophy, as reported in a group of patients with a 22q11.2 deletion syndrome [86]. It is tempting to speculate that Glu levels alone may not capture early disruptions in the excitation/inhibition balance, but that, with illness progression, this imbalance becomes more prominent and manifests as a measurable elevation in Glu.

#### Strengths and limitations

This study’s major strengths include a large cohort of medication-naïve FEP and matched controls, and a prospective longitudinal design over three time points allowing us to identify potential inflexion time points. As previously mentioned, we did not use FreeSurfer’s longitudinal pipeline. This is a potential limitation because the longitudinal pipeline registers each image to a temporally neutral base template before segmentation, which together with other techniques effectively reduces post-processing noise in subsequent results. However, this longitudinal pipeline does not support the addition of T2-weighted images when segmenting out subfield regions, which has been shown to increase subfield segmentation accuracy [51]. To help compensate for this we analyzed cross-sectional data using linear mixed models.

While exposure to cannabis may affect brain structure and biochemistry, it is considered one of the major risk factors for developing psychosis and consequently highly clinically relevant. Excluding patients using cannabis would have inadvertently biased our sample and limited the generalizability of our results. It should be noted that over the course of our data collection there was one system update to the scanner used to collect this data. This may have been a potential confound that we did not control for in our analysis. Additionally, it should be noted that we did not control for race/ethnicity within our analysis. Little is known about the effect that racial/ethnicity differences might have on brain imaging, but future studies should specifically address this issue.

## Conclusions

These findings add to the existing literature by showing that initially localized hippocampal volume deficits in medication naïve FEP continue to expand throughout the hippocampus even after 16 weeks of APD treatment. Furthermore, this process does not appear to be associated with glutamate levels prior to or after APD treatment.

## Supplementary information

SUPPLEMENTAL MATERIAL

## Data Availability

The dataset here has subject overlap with our previous studies [2, 7, 87, 88]. Data for NCT 034420101 is deposited in the NDA data archive and shared per NIMH agreement.
