# Supplementary material for: A longitudinal study of hippocampal subfield volumes and hippocampal glutamate levels in antipsychotic-naïve first episode psychosis patients
Source: Mol Psychiatry. 2024 Nov 23;30(5):2017–26. doi: 10.1038/s41380-024-02812-1 (PMC12014507; doi:10.1038/s41380-024-02812-1)
Supplement: Supplementary file 1 — SUPPLEMENTAL MATERIAL [file 41380_2024_2812_MOESM1_ESM.docx]

# SUPPLEMENTAL

### *Participants Per Analysis*

Because mixed models do not remove participants with missing data points our full participant count for our initial volume based mixed models were 93 FEP and 80 HC, however the number of participants included at each time point varied. At baseline 1 FEP was excluded due to poor scan quality, while 1 HC lacked a baseline scan, leaving a total of 92 FEP and 79 HC included baseline. At week 6, 1 FEP was excluded for being unmedicated, another FEP was excluded for poor scan quality and 19 FEP lacked week 6 scans, while 23 HC lacked week 6 scans, leaving our week 6 scan total at 72 FEP and 57 HC. Finally, at week 16, 5 FEP were unmedicated and thus excluded from week 16 and another 24 FEP lacked week 16 scans, while 28 HC lacked week 16 scans. This left our week 16 scan count at 64 FEP and 52 HC.

88 FEP and 77 HC had MRS data at one or more timepoints and were included in our initial Glu mixed models. Again, the number of participants included at each time point varied. 7 FEP and 4 HC did not have baseline MRS data. 1 FEP was excluded due to FWHM, and another 6 FEP and 2 HC were excluded for CRLB, leaving 74 FEP and 71 HC baseline participants for analysis. At week 6, 15 FEP and 26 HC did not have MRS data. 1 FEP was excluded due to FWHM, another 6 FEP and 2 HC excluded for CRLB. This left 66 FEP and 49 HC at week 6 for analysis. Lastly at week 16, 21 FEP and 25 HC did not have MRS data. 2 FEP were excluded due to FWHM, 6 FEP had Glu data excluded for CRLB. This left 56 FEP and 52 HC at week 16 for analysis. Inclusion of participants for the correlation analysis to determine if baseline Glu was associated with baseline volumes or if it was predictive of volume change required that participants had both baseline volumes and baseline glutamate and volume measures at both baseline and week 16 in addition to baseline Glu measures respectively. Because of this only 71 FEP and 74 HC could be included to assess correlations between baseline volumes and baseline Glu, while only 51 FEP and 45 HC could be included to test Glu if glutamate was a predictor of change in volumes. All of this is shown visually in figure 1

| **Table S1: Mixed Model F-test results for Bilateral Subfield and Bilateral Hippocampus Volumes** | | | | | | | | | | |
| --- | --- | --- | --- | --- | --- | --- | --- | --- | --- | --- |
|  | CA1 | | CA3 | | CA4 | | Subiculum | | Presubiculum | |
| Fixed Effects | *F* (df1, df2) | *p* | *F* (df1, df2) | *p* | *F* (df1, df2) | *p* | *F* (df1, df2) | *p* | *F* (df1, df2) | *p* |
| Age | 0.04 (1, 157.2) | 0.8401 | 0.17 (1, 166.4) | 0.6797 | 0.09 (1, 160.2) | 0.7709 | 0.22 (1, 161.8) | 0.6394 | 0.16 (1, 165.3) | 0.6865 |
| ICV | 45.13 (1, 315.0) | **<0.0001** | 48.47 (1, 277.3) | **<.0001** | 52.22 (1, 257.3) | **<.0001** | 48.66 (1, 260.4) | **<.0001** | 81.39 (1, 253.7) | **<.0001** |
| **Time** | 5.64 (2, 229.7) | **0.0041** | 1.07 (2, 240.7) | 0.1375 | 3.25 (2, 235.1) | **0.0405** | 4.02 (2, 239.0) | **0.0191** | 4.29 (2, 240.9) | **0.0148** |
| **Group** | 14.34 (1, 159.4) | **0.0002** | 2.00 (1, 169.5) | 0.3032 | 6.61 (1, 163.5) | **0.0110** | 14.86 (1, 163.3) | **0.0002** | 15.46 (1, 169.0) | **0.0001** |
| **Group X Time** | 4.58 (2, 229.8) | **0.0013** | 5.52 (2, 240.8) | **0.0045** | 4.84 (2, 235.2) | **0.0087** | 1.03 (2, 237.0) | 0.3597 | 9.34 (2, 241.0) | **0.0001** |

| **Table S1: Continued** | | | | | | | | |
| --- | --- | --- | --- | --- | --- | --- | --- | --- |
|  | Molecular Layer | | GC/ML/DG | | Hippocampal Tail | | Whole Hippocampus | |
| Fixed Effects | *F* (df1, df2) | *p* | *F* (df1, df2) | *p* | *F* (df1, df2) | *p* | *F* (df1, df2) | *p* |
| Age | 1.60 (1, 162.4) | 0.2080 | 0.09 (1, 160.7) | 0.7621 | <0.00 (1, 168.0) | 0.9541 | <0.00 (1,157.0) | 0.9513 |
| ICV | 81.57 (1, 209.8) | **<.0001** | 55.75 (1, 249.8) | **<.0001** | 35.15 (1, 288.4) | **<.0001** | 61.69 (1, 340.7) | **<.0001** |
| **Time** | 4.67 (2, 245.0) | **0.0103** | 2.76 (2, 236.2) | 0.0651 | 1.56 (2, 244.2) | 0.2130 | 4.64 (2, 228.8) | **0.0106** |
| **Group** | 4.18 (1, 165.0) | **0.0425** | 0.09 (1, 164.4) | **0.0038** | <0.00 (1, 169.3) | **0.0095** | 12.96 (1, 159.4) | **0.0004** |
| **Group x Time** | 0.62 (2, 243.3) | 0.5352 | 4.38 (2, 236.3) | **0.0136** | 1.71 (2, 241.9) | 0.1834 | 5.81 (2, 228.9) | **0.0035** |
| ICV, intracranial volume. | | | | | | | | |

| **Table S2: Post Hoc Comparisons for Hippocampal and Subfield Volumes** | | | | | | | | | |
| --- | --- | --- | --- | --- | --- | --- | --- | --- | --- |
|  |  |  |  | **CA1** | **CA3** | **CA4** | **Presubiculum** | **GC/ML/DG** | **Total Hippocampus** |
| Between Group Comparisons | | | | *p* | *p* | *p* | *p* | *p* | *p* |
|  | Baseline | | | **0.0165** | 0.9662 | 0.4721 | **0.0009** | 0.2358 | **0.0202** |
|  | Week 6 | | | **0.0104** | 0.9991 | 0.2707 | 0.0510 | 0.1559 | **0.0237** |
|  | Week 16 | | | **0.0002** | 0.4749 | **0.0089** | **0.0003** | **0.0029** | **0.0004** |
| Within Groups Comparisons | | | |  |  |  |  |  |  |
| HC |  |  |  |  |  |  |  |  |  |
|  | Baseline - Week 6 | | | 0.7406 | 0.7113 | 0.9938 | **0.0015** | 0.9861 | 0.8121 |
|  | Baseline - Week 16 | | | >0.9999 | >0.9999 | 0.9980 | 0.8541 | 0.9992 | >0.9999 |
|  | Week 6 - Week 16 | | | 0.9814 | 0.9479 | 0.9558 | 0.7988 | 0.9590 | 0.9863 |
| FEP |  |  |  |  |  |  |  |  |  |
|  | Baseline - Week 6 | | | 0.1448 | 0.9973 | 0.2277 | 0.9947 | 0.5427 | 0.8723 |
|  | Baseline - Week 16 | | | **0.0002** | **0.0464** | **0.0009** | 0.1420 | **0.0025** | **0.0003** |
|  | Week 6 - Week 16 | | | **0.0053** | **0.0035** | **0.0152** | **0.0177** | **0.0139** | **0.0002** |
| HC, healthy control; FEP, first episode psychosis patient.  Covariates included age and intracranial volume. *p* value corrected for multiple comparisons using Tukey’s HSD. | | | | | | | | | |

| **Table S3: Mixed Model F-test results for Hippocampal Glutamate** | | |
| --- | --- | --- |
| Fixed Effects | *F* (df1, df2) | *p* |
| Sex | 6.18 (1, 185.4) | **0.0138** |
| PPD | 0.44 (1, 192.2) | 0.5094 |
| FWHM | 31.03 (1, 344.2) | **<.0001** |
| **Time** | 0.78 (2, 246.6) | 0.4594 |
| **Group** | <0.01 (1, 183.1) | 0.9663 |
| **Group x Time** | 4.60 (2, 246.9) | **0.0109** |
| PPD, packs per day; FWHM, full-width at half maximum. | | |

| **Table S4: Post Hoc Comparisons for Hippocampal Glutamate** | | | | |
| --- | --- | --- | --- | --- |
| Between Group Comparisons | | | | *p* |
|  | Baseline | | | 0.8699 |
|  | Week 6 | | | 0.2539 |
|  | Week 16 | | | 0.8311 |
| Within Groups Comparisons | | | |  |
| HC |  |  |  |  |
|  | Baseline - Week 6 | | | 0.3756 |
|  | Baseline - Week 16 | | | 0.9545 |
|  | Week 6 - Week 16 | | | 0.1314 |
| FEP |  |  |  |  |
|  | Baseline - Week 6 | | | 0.9884 |
|  | Baseline - Week 16 | | | 0.9881 |
|  | Week 6 - Week 16 | | | 0.9258 |
| HC, healthy control; FEP, first episode psychosis patient.  Covariates included sex, smoking status (packs per day), and FWHM. *p* value corrected for multiple comparisons using Tukey’s HSD. | | | | |

| **Table S5: Glutamate and Volume Correlations Healthy Controls (uncorrected p-values)** | | | | | | |
| --- | --- | --- | --- | --- | --- | --- |
| **Baseline Glutamate x Baseline Volume** | | | | | | |
|  | **CA1** | **CA3** | **CA4** | **Presub** | **GC/ML/DG** | **Total Hipp** |
| ***r*** | 0.1256 | 0.1275 | 0.1438 | 0.0862 | 0.1377 | 0.1272 |
| **95%CI** | -0.1120 0.3478 | -0.1142 0.3458 | -0.0960 0.3619 | -0.1361 0.3261 | -0.1019 0.3567 | -0.1058 0.3532 |
| ***p*** | 0.3007 | 0.3092 | 0.2427 | 0.4047 | 0.2631 | 0.2773 |
| **Baseline Glutamate x Change in Volume (Week 16 – Baseline)** | | | | | | |
| ***r*** | 0.0958 | -0.0670 | -0.0760 | -0.0896 | -0.0622 | -0.0978 |
| **95%CI** | -0.2034 0.3787 | -0.3536 0.2311 | -0.3615 0.2225 | -0.3734 0.2094 | -0.3493 0.2357 | -0.3804 0.2016 |
| ***p*** | 0.5312 | 0.6620 | 0.6197 | 0.5581 | 0.6849 | 0.5229 |
| p values are uncorrected. Because initial regression models were not significant there was no reason to adjust for covariates. 95% confidence intervals, low (top) to high (bottom) range | | | | | | |
|  | | | | | | |

| **Table S6: Correlation of Glutamate with BPRS scores and Treatment Response** | | | | | | | | | |
| --- | --- | --- | --- | --- | --- | --- | --- | --- | --- |
|  | | df | *r* | *p* |  | | df | *r* | *p* |
| Baseline Glutamate x Baseline BPRS | | | | | Week 16 Glutamate x Week 16 BPRS | | | | |
|  | Total | 70 | 0.001 | 0.991 |  | Total | 51 | -0.017 | 0.902 |
|  | Positive | 70 | 0.049 | 0.681 |  | Positive | 51 | 0.168 | 0.228 |
|  | Negative | 70 | -0.014 | 0.909 |  | Negative | 51 | -0.103 | 0.463 |
| Baseline Glutamate x Treatment Response | | | | | | | | | |
|  | | 53 | 0.016 | 0.905 |  | |  |  |  |
| BPRS, Brief Psychiatric Rating Scale (positive subscale included conceptual disorganization, hallucinatory behavior, and unusual thought content; negative subscale included emotional withdrawal, motor retardation, and blunted affect)  Treatment response, % change in BPRS positive score from [A] baseline to [B] week 16: (((B‐A)/A)*–100)  Covariates included sex and smoking status (packs per day) | | | | | | | | | |

| **Table S7: Correlation of Hippocampal Subfields with BPRS scores and Treatment Response** | | | | | | | |
| --- | --- | --- | --- | --- | --- | --- | --- |
|  | | Baseline BPRS Scale | | | | | |
|  |  | Total | | Positive | | Negative | |
| Baseline Subfields | df | *r* | *p* | *r* | *p* | *r* | *p* |
| CA1 | 88 | 0.003 | 0.981 | 0.037 | 0.728 | 0.027 | 0.797 |
| CA3 | 88 | -0.041 | 0.702 | -0.027 | 0.8 | 0.008 | 0.943 |
| CA4 | 88 | -0.025 | 0.818 | 0.002 | 0.985 | 0.073 | 0.494 |
| Subiculum | 88 | 0.049 | 0.646 | 0.095 | 0.375 | 0.071 | 0.508 |
| Presubiculum | 88 | 0.078 | 0.467 | 0.149 | 0.16 | 0.005 | 0.959 |
| Molecular Layer | 88 | -0.001 | 0.994 | 0.035 | 0.745 | 0.005 | 0.960 |
| GC/ML/DG | 88 | -0.005 | 0.960 | 0.028 | 0.796 | 0.085 | 0.426 |
| Hippocampal Tail | 88 | 0.145 | 0.174 | 0.156 | 0.141 | 0.043 | 0.684 |
|  | | | | | | | |
|  | | Week 16 BPRS Scale | | | | | |
|  |  | Total | | Positive | | Negative | |
| Week 16 Subfields | df | *r* | *p* | *r* | *p* | *r* | *p* |
| CA1 | 60 | -0.076 | 0.558 | -0.081 | 0.529 | 0.054 | 0.676 |
| CA3 | 60 | 0.046 | 0.725 | -0.150 | 0.245 | 0.226 | 0.077 |
| CA4 | 60 | 0.041 | 0.754 | -0.114 | 0.380 | 0.249 | 0.051 |
| Subiculum | 60 | 0.012 | 0.928 | -0.037 | 0.778 | 0.085 | 0.510 |
| Presubiculum | 60 | -0.038 | 0.771 | 0.072 | 0.580 | 0.005 | 0.969 |
| Molecular Layer | 60 | 0.044 | 0.733 | -0.103 | 0.427 | 0.165 | 0.201 |
| GC/ML/DG | 60 | 0.027 | 0.835 | -0.129 | 0.316 | 0.268 | **0.035** |
| Hippocampal Tail | 60 | 0.174 | 0.175 | -0.057 | 0.659 | 0.258 | **0.043** |
|  |  | | |  | |  | |
|  | Treatment Response | | |  | |  | |
| Baseline Subfields | df | *r* | *p* |  |  |  |  |
| CA1 | 65 | -0.137 | 0.268 |  |  |  |  |
| CA3 | 65 | -0.156 | 0.208 |  |  |  |  |
| CA4 | 65 | -0.240 | 0.050 |  |  |  |  |
| Subiculum | 65 | -0.176 | 0.155 |  |  |  |  |
| Presubiculum | 65 | -0.108 | 0.386 |  |  |  |  |
| Molecular Layer | 65 | -0.047 | 0.706 |  |  |  |  |
| GC/ML/DG | 65 | -0.268 | **0.029** |  |  |  |  |
| Hippocampal Tail | 65 | -0.101 | 0.415 |  |  |  |  |
| BPRS, Brief Psychiatric Rating Scale (positive subscale included conceptual disorganization, hallucinatory behavior, and unusual thought content; negative subscale included emotional withdrawal, motor retardation, and blunted affect);  Treatment response, % change in BPRS positive score from [A] baseline to [B] week 16: (((B‐A)/A)*–100)  Covariates included intracranial volume and age | | | | | | | |

| **Table S8A: RBANS Association with Baseline Hippocampal Subfield Volumes and Prediction of Week 16 Hippocampal Volumes in FEP** | | | | | | | | | | | | | | |
| --- | --- | --- | --- | --- | --- | --- | --- | --- | --- | --- | --- | --- | --- | --- |
|  | | | RBANS | | | | | | | | | | | |
|  | |  | Total | | Immediate Memory | | Visuospatial | | Language | | Attention | | Delayed Memory | |
| Baseline Subfields | | df | *r* | *p* | *r* | *p* | *r* | *p* | *r* | *p* | *r* | *p* | *r* | *p* |
|  | CA1 | 77 | 0.096 | 0.399 | -0.054 | 0.634 | 0.160 | 0.160 | 0.143 | 0.208 | 0.059 | 0.605 | 0.059 | 0.608 |
|  | CA3 | 77 | 0.052 | 0.652 | -0.038 | 0.740 | 0.073 | 0.521 | 0.043 | 0.705 | 0.024 | 0.835 | 0.070 | 0.539 |
|  | CA4 | 77 | 0.113 | 0.323 | -0.005 | 0.966 | 0.137 | 0.230 | 0.113 | 0.320 | 0.053 | 0.643 | 0.090 | 0.428 |
|  | Subiculum | 77 | 0.050 | 0.663 | -0.029 | 0.798 | 0.037 | 0.745 | 0.054 | 0.638 | 0.063 | 0.583 | 0.116 | 0.310 |
|  | Presubiculum | 77 | -0.048 | 0.676 | -0.115 | 0.313 | -0.033 | 0.774 | 0.050 | 0.659 | 0.006 | 0.955 | -0.016 | 0.886 |
|  | Molecular Layer | 77 | 0.091 | 0.424 | -0.047 | 0.678 | 0.228 | **0.043** | 0.043 | 0.709 | 0.022 | 0.846 | 0.086 | 0.450 |
|  | GC/ML/DG | 77 | 0.107 | 0.348 | -0.022 | 0.845 | 0.132 | 0.246 | 0.111 | 0.328 | 0.068 | 0.552 | 0.083 | 0.468 |
|  | Hippocampal Tail | 77 | 0.005 | 0.968 | -0.119 | 0.296 | 0.004 | 0.972 | 0.020 | 0.859 | 0.015 | 0.893 | 0.125 | 0.272 |
|  | | | | | | | | | | | | | | |
| Week 16 Subfields | |  |  |  |  |  |  |  |  |  |  |  |  |  |
|  | CA1 | 58 | 0.000 | 0.998 | -0.199 | 0.128 | 0.059 | 0.656 | 0.054 | 0.684 | 0.070 | 0.593 | -0.002 | 0.988 |
|  | CA3 | 58 | -0.072 | 0.585 | -0.259 | **0.046** | -0.033 | 0.800 | -0.069 | 0.602 | 0.027 | 0.836 | -0.003 | 0.985 |
|  | CA4 | 58 | -0.002 | 0.988 | -0.217 | 0.096 | 0.033 | 0.803 | -0.021 | 0.876 | 0.096 | 0.466 | 0.020 | 0.879 |
|  | Subiculum | 58 | -0.009 | 0.945 | -0.183 | 0.161 | 0.073 | 0.581 | -0.036 | 0.784 | 0.066 | 0.615 | 0.060 | 0.650 |
|  | Presubiculum | 58 | -0.011 | 0.932 | -0.229 | 0.078 | 0.095 | 0.472 | 0.041 | 0.757 | 0.066 | 0.615 | 0.043 | 0.742 |
|  | Molecular Layer | 58 | -0.148 | 0.258 | -0.336 | **0.009** | 0.005 | 0.971 | -0.139 | 0.289 | -0.066 | 0.618 | -0.067 | 0.611 |
|  | GC/ML/DG | 58 | 0.005 | 0.971 | -0.233 | 0.073 | 0.048 | 0.717 | -0.006 | 0.961 | 0.104 | 0.430 | 0.033 | 0.803 |
|  | Hippocampal Tail | 58 | -0.062 | 0.636 | -0.246 | 0.058 | 0.023 | 0.861 | -0.040 | 0.762 | -0.040 | 0.762 | 0.075 | 0.568 |
| RBANS, Repeatable Battery for Assessment of Neuropsychological.  Covariates included intracranial volume and age | | | | | | | | | | | | | | |

| **Table S8B: RBANS Association with Baseline Hippocampal Subfield Volumes and Prediction of Week 16 Hippocampal Volumes in HC** | | | | | | | | | | | | | | |
| --- | --- | --- | --- | --- | --- | --- | --- | --- | --- | --- | --- | --- | --- | --- |
|  | | | RBANS | | | | | | | | | | | |
|  | |  | Total | | Immediate Memory | | Visuospatial | | Language | | Attention | | Delayed Memory | |
| Baseline Subfields | | df | *r* | *p* | *r* | *p* | *r* | *p* | *r* | *p* | *r* | *p* | *r* | *p* |
|  | CA1 | 64 | 0.176 | 0.158 | 0.160 | 0.200 | 0.098 | 0.434 | 0.137 | 0.273 | 0.066 | 0.600 | 0.066 | 0.599 |
|  | CA3 | 64 | 0.185 | 0.137 | 0.261 | **0.034** | 0.040 | 0.752 | 0.075 | 0.547 | 0.065 | 0.602 | 0.074 | 0.556 |
|  | CA4 | 64 | 0.175 | 0.160 | 0.177 | 0.155 | 0.105 | 0.402 | 0.076 | 0.544 | 0.068 | 0.589 | 0.100 | 0.426 |
|  | Subiculum | 64 | 0.061 | 0.626 | 0.009 | 0.943 | 0.171 | 0.170 | 0.020 | 0.872 | 0.023 | 0.854 | -0.032 | 0.802 |
|  | Presubiculum | 64 | 0.162 | 0.193 | -0.001 | 0.995 | 0.180 | 0.147 | 0.133 | 0.287 | 0.134 | 0.283 | 0.023 | 0.858 |
|  | Molecular Layer | 64 | 0.047 | 0.709 | 0.044 | 0.724 | 0.065 | 0.604 | 0.057 | 0.647 | 0.003 | 0.981 | -0.064 | 0.609 |
|  | GC/ML/DG | 64 | 0.178 | 0.154 | 0.171 | 0.171 | 0.103 | 0.409 | 0.063 | 0.615 | 0.090 | 0.470 | 0.118 | 0.346 |
|  | Hippocampal Tail | 64 | 0.156 | 0.210 | 0.123 | 0.325 | 0.149 | 0.232 | 0.071 | 0.571 | 0.050 | 0.691 | 0.044 | 0.728 |
|  | | | | | | | | | | | | | | |
| Week 16 Subfields | |  |  |  |  |  |  |  |  |  |  |  |  |  |
|  | CA1 | 45 | 0.155 | 0.298 | 0.059 | 0.694 | 0.206 | 0.165 | 0.084 | 0.573 | 0.056 | 0.707 | 0.105 | 0.481 |
|  | CA3 | 45 | 0.150 | 0.314 | 0.133 | 0.374 | 0.128 | 0.390 | 0.068 | 0.648 | 0.020 | 0.893 | 0.123 | 0.412 |
|  | CA4 | 45 | 0.094 | 0.529 | 0.007 | 0.964 | 0.193 | 0.193 | 0.021 | 0.888 | 0.011 | 0.941 | 0.088 | 0.555 |
|  | Subiculum | 45 | -0.041 | 0.783 | -0.186 | 0.211 | 0.145 | 0.330 | -0.093 | 0.534 | 0.075 | 0.616 | -0.054 | 0.717 |
|  | Presubiculum | 45 | 0.113 | 0.448 | -0.120 | 0.423 | 0.133 | 0.374 | 0.034 | 0.820 | 0.212 | 0.152 | 0.120 | 0.422 |
|  | Molecular Layer | 45 | -0.016 | 0.917 | -0.048 | 0.749 | 0.074 | 0.623 | -0.014 | 0.924 | -0.019 | 0.897 | -0.045 | 0.763 |
|  | GC/ML/DG | 45 | 0.079 | 0.599 | -0.014 | 0.924 | 0.179 | 0.229 | 0.001 | 0.996 | 0.045 | 0.764 | 0.065 | 0.663 |
|  | Hippocampal Tail | 45 | 0.110 | 0.462 | 0.004 | 0.978 | 0.279 | 0.057 | -0.042 | 0.780 | 0.058 | 0.696 | 0.044 | 0.770 |
| RBANS, Repeatable Battery for Assessment of Neuropsychological.  Covariates included intracranial volume and age | | | | | | | | | | | | | | |

| **Table S9: Correlation of** **Duration of Untreated Psychosis with RBANS** | | | |
| --- | --- | --- | --- |
|  | df | *r* | *p* |
| RBANS |  |  |  |
| Total | 80 | 0.006 | 0.960 |
| Immediate Memory | 80 | -0.150 | 0.178 |
| Visuospatial | 80 | 0.054 | 0.630 |
| Language | 80 | 0.103 | 0.359 |
| Attention | 80 | 0.056 | 0.616 |
| Delayed Memory | 80 | -0.031 | 0.782 |
| RBANS, Repeatable Battery for Assessment of Neuropsychological. | | | |

| **Table S10: Correlation of RBANS and RBANS Subscales with Baseline and Week 16 BPRS and BPRS Subscales and Treatment Response** | | | | | | | | | |
| --- | --- | --- | --- | --- | --- | --- | --- | --- | --- |
|  |  |  | Baseline BPRS | | | | | |  |
|  |  |  | Total | | Positive | | Negative | |  |
|  |  | df | *r* | *p* | *r* | *p* | *r* | *p* |  |
| RBANS | |  |  |  |  |  |  |  |  |
|  | Total | 80 | -0.094 | 0.402 | -0.072 | 0.520 | -0.065 | 0.561 |  |
|  | Immediate Memory | 80 | -0.043 | 0.704 | -0.068 | 0.543 | -0.019 | 0.866 |  |
|  | Visuospatial | 80 | -0.048 | 0.669 | -0.065 | 0.559 | 0.008 | 0.941 |  |
|  | Language | 80 | -0.215 | 0.052 | -0.122 | 0.276 | -0.068 | 0.543 |  |
|  | Attention | 80 | -0.051 | 0.652 | -0.025 | 0.823 | -0.110 | 0.324 |  |
|  | Delayed Memory | 80 | 0.005 | 0.961 | 0.017 | 0.877 | -0.067 | 0.551 |  |
|  |  |  |  | | | | | |  |
|  |  |  | Week 16 BPRS | | | | | |  |
|  | Total | 65 | -0.177 | 0.152 | -0.178 | 0.150 | -0.072 | 0.565 |  |
|  | Immediate Memory | 65 | -0.241 | **0.049** | -0.207 | 0.093 | -0.074 | 0.553 |  |
|  | Visuospatial | 65 | 0.004 | 0.974 | -0.080 | 0.521 | -0.006 | 0.960 |  |
|  | Language | 65 | -0.224 | 0.069 | -0.068 | 0.584 | -0.143 | 0.248 |  |
|  | Attention | 65 | 0.025 | 0.843 | 0.034 | 0.782 | -0.070 | 0.571 |  |
|  | Delayed Memory | 65 | -0.268 | **0.028** | -0.337 | **0.005** | -0.027 | 0.829 |  |
|  |  |  |  | | |  |  |  |  |
|  |  |  | Treatment Response | | |  |  |  |  |
|  | Total | 65 | -0.074 | 0.553 |  |  |  |  |  |
|  | Immediate Memory | 65 | -0.057 | 0.648 |  |  |  |  |  |
|  | Visuospatial | 65 | 0.029 | 0.814 |  |  |  |  |  |
|  | Language | 65 | -0.084 | 0.501 |  |  |  |  |  |
|  | Attention | 65 | 0.010 | 0.936 |  |  |  |  |  |
|  | Delayed Memory | 65 | -0.175 | 0.157 |  |  |  |  |  |
| RBANS, Repeatable Battery for Assessment of Neuropsychological; BPRS, Brief Psychiatric Rating Scale (positive subscale included conceptual disorganization, hallucinatory behavior, and unusual thought content; negative subscale included emotional withdrawal, motor retardation, and blunted affect).  Treatment response, % change in BPRS positive score from [A] baseline to [B] week 16: (((B‐A)/A)*–100). | | | | | | | | | |

| **Table S11: Correlation of Subfields with Duration of Untreated Psychosis** | | | | | | |
| --- | --- | --- | --- | --- | --- | --- |
|  | Baseline | | | Week 16 | | |
| Subfield | df | *r* | *p* | df | *r* | *p* |
| CA1 | 88 | -0.123 | 0.246 | 60 | 0.012 | 0.927 |
| CA3 | 88 | -0.109 | 0.309 | 60 | 0.033 | 0.799 |
| CA4 | 88 | -0.098 | 0.358 | 60 | 0.029 | 0.825 |
| Subiculum | 88 | -0.218 | **0.039** | 60 | -0.032 | 0.804 |
| Presubiculum | 88 | -0.176 | 0.097 | 60 | -0.022 | 0.863 |
| Molecular Layer | 88 | -0.121 | 0.255 | 60 | 0.180 | 0.160 |
| GC/ML/DG | 88 | -0.084 | 0.432 | 60 | 0.021 | 0.872 |
| Hippocampal Tail | 88 | -0.210 | **0.046** | 60 | -0.161 | 0.210 |
| Covariates included intracranial volume and age | | | | | | |

| **Table S12: Mixed Model F-test results for Bilateral Subfield and Bilateral Hippocampus Volumes**  **(Completers, N: FEP = 35, HC = 31)** | | | | | | | | | | |
| --- | --- | --- | --- | --- | --- | --- | --- | --- | --- | --- |
|  | CA1 | | CA3 | | CA4 | | Subiculum | | Presubiculum | |
| Fixed Effects | *F* (df1, df2) | *p* | *F* (df1, df2) | *p* | *F* (df1, df2) | *p* | *F* (df1, df2) | *p* | *F* (df1, df2) | *p* |
| Age | 0.48 (1, 53.4) | 0.4923 | 1.31 (1, 63.6) | 0.2558 | 1.79 (1, 60.6) | 0.1863 | 2.00 (1, 53.8) | 0.1632 | 2.25 (1, 60.6) | 0.1387 |
| ICV | 13.44 (1, 151.2) | **0.0003** | 26.43 (1, 119.2) | **<.0001** | 28.70 (1,116.7) | **<.0001** | 13.29 (1, 108.9) | **0.0004** | 42.87 (1, 106.8) | **<.0001** |
| **Time** | 1.83 (2, 116.9) | 0.1649 | 1.45 (2, 128.1) | 0.2380 | 3.23 (2, 125.1) | **0.0431** | 2.91 (2, 117.6) | 0.0584 | 4.19 (2, 125.2) | **0.0174** |
| **Group** | 3.91 (1, 53.3) | 0.0531 | 0.51 (1, 63.8) | 0.4770 | 1.12 (1, 60.8) | 0.2935 | 4.53 (1, 54.0) | **0.0378** | 8.82 (1, 61.0) | **0.0042** |
| **Group X Time** | 2.69 (2, 116.9) | 0.0723 | 4.89 (2, 128.1) | **0.0090** | 3.16 (2, 125.1) | **0.0458** | 1.80 (2, 117.6) | 0.1699 | 2.12 (2, 125.2) | 0.1239 |

| **Table S12: Continued** | | | | | | | | |
| --- | --- | --- | --- | --- | --- | --- | --- | --- |
|  | Molecular Layer | | GC/ML/DG | | Hippocampal Tail | | Whole Hippocampus | |
| Fixed Effects | *F* (df1, df2) | *p* | *F* (df1, df2) | *p* | *F* (df1, df2) | *p* | *F* (df1, df2) | *p* |
| Age | 4.16 (1, 63.7) | 0.0455 | 2.16 (1, 61.5) | 0.1465 | 2.00 (1, 63.2) | 0.1626 | 1.49 (1, 55.9) | 0.2275 |
| ICV | 26.06 (1, 88.3) | **<.0001** | 29.86 (1, 110.9) | **<.0001** | 13.40 (1, 133.4) | **0.0004** | 24.54 (1, 165.8) | **<.0001** |
| **Time** | 2.24 (2, 128.1) | 0.1107 | 2.12 (2, 126.1) | 0.1239 | 2.30 (2, 127.6) | 0.1043 | 3.23 (2, 119.7) | **0.0432** |
| **Group** | 2.03 (1, 64.5) | 0.1594 | 1.44 (1, 61.8) | 0.2341 | 2.08 (1, 63.3) | 0.1542 | 4.51 (1, 56.3) | **0.0382** |
| **Group x Time** | 0.30 (2, 128.1) | 0.7431 | 3.29 (2, 126.1) | **0.0404** | 1.37 (2, 127.6) | 0.2586 | 3.76 (2, 119.7) | **0.0261** |
| Completers, subjects without missing data points for mixed model analyses; ICV, intracranial volume. | | | | | | | | |

| **Table S13: Post Hoc Comparisons for Hippocampal and Subfield Volumes (Completers)** | | | | | | |
| --- | --- | --- | --- | --- | --- | --- |
|  | | **CA3** | **CA4** | **Presubiculum** | **GC/ML/DG** | **Total Hippocampus** |
| Between Group Comparisons | | *p* | *p* | *p* | *p* | *p* |
|  | Baseline | 0.9610 | 0.7273 | **0.0425** | 0.9581 | 0.4231 |
|  | Week 6 | 1.0000 | 1.0000 | 0.1375 | 0.9745 | 0.4575 |
|  | Week 16 | 0.8828 | 0.9537 | **0.0235** | 0.4160 | 0.1077 |
| Within Groups Comparisons | |  |  |  |  |  |
| HC | N = 31 |  |  |  |  |  |
|  | Baseline - Week 6 | 0.2063 | 0.9867 | 0.1304 | 0.8182 | 0.8851 |
|  | Baseline - Week 16 | 0.9644 | 0.9820 | 0.5541 | 1.0000 | 0.9996 |
|  | Week 6 - Week 16 | 0.9867 | 0.5100 | 0.9999 | 0.9629 | 0.9989 |
| FEP | N = 35 |  |  |  |  |  |
|  | Baseline - Week 6 | 0.9536 | 0.5432 | 0.9956 | 0.9527 | 0.9795 |
|  | Baseline - Week 16 | 0.5896 | 0.0177 | 0.1942 | 0.0513 | **0.0132** |
|  | Week 6 - Week 16 | 0.1091 | 0.0758 | 0.1527 | 0.0546 | **0.0065** |
| HC, healthy control; FEP, first episode psychosis patient.  Covariates included age and intracranial volume. p value corrected for multiple comparisons using Tukey’s HSD. | | | | | | |

| **Table S15: Post Hoc Comparisons for Hippocampal Glutamate (Completers)** | | | | |
| --- | --- | --- | --- | --- |
| Between Group Comparisons | | | | *p* |
|  | Baseline | | | 0.9945 |
|  | Week 6 | | | 0.1140 |
|  | Week 16 | | | 0.6083 |
| Within Groups Comparisons | | | |  |
| HC |  |  |  |  |
|  | Baseline - Week 6 | | | 0.6483 |
|  | Baseline - Week 16 | | | 0.5332 |
|  | Week 6 - Week 16 | | | **0.0253** |
| FEP |  |  |  |  |
|  | Baseline - Week 6 | | | 0.3720 |
|  | Baseline - Week 16 | | | 0.9817 |
|  | Week 6 - Week 16 | | | 0.8788 |
| HC, healthy control; FEP, first episode psychosis patient.  Covariates included sex, smoking status (packs per day), and FWHM. p value corrected for multiple comparisons using Tukey’s HSD. | | | | |

| **Table S14: Mixed Model F-test results for Hippocampal Glutamate (Completers, N: FEP = 35, HC = 31 )** | | |
| --- | --- | --- |
| Fixed Effects | *F* (df1, df2) | *p* |
| Sex | 9.07 (1, 79.7) | **0.0035** |
| PPD | 0.01 (1, 79.9) | 0.9239 |
| FWHM | 14.83 (1, 173.4) | **0.0002** |
| **Time** | 1.68 (2, 113.1) | 0.1912 |
| **Group** | 0.05 (1, 75.9) | 0.8196 |
| **Group x Time** | 5.32 (2, 114.3) | **0.0062** |
| Completers, subjects without missing data points for mixed model analyses; PPD, packs per day; FWHM, full-width at half maximum. | | |

| **Table S17: Subgroup Post Hoc Comparisons for Hippocampal and Subfield Volumes** | | | | | | | | | |
| --- | --- | --- | --- | --- | --- | --- | --- | --- | --- |
|  | | | **CA1** | **CA4** | **Subiculum** | **Presubiculum** | **Mol. Layer** | **GC/ML/DG** | **Total Hippocampus** |
| Between Group Comparisons | | | *p* | *p* | *p* | *p* | *p* | *p* | *p* |
|  | Baseline | | 0.9750 | 0.9999 | 0.3432 | 0.3086 | 0.4119 | 0.9999 | 0.9108 |
|  | Week 6 | | 0.8681 | 0.9893 | 0.1298 | 0.2697 | 0.1289 | 0.9843 | 0.5954 |
|  | Week 16 | | 0.9424 | 0.9998 | 0.5207 | 0.7492 | 0.6890 | 0.9999 | 0.8950 |
| Within Groups Comparisons | | |  |  |  |  |  |  |  |
| SZ-FEP | | N = 48 |  |  |  |  |  |  |  |
|  | Baseline - Week 6 | | 0.1160 | 0.0315 | 0.1106 | 0.9999 | 0.9999 | 0.0675 | 0.2892 |
|  | Baseline - Week 16 | | **0.0106** | **0.0121** | 0.5467 | 0.8384 | 1.0000 | **0.0212** | **0.0421** |
|  | Week 6 - Week 16 | | 0.2067 | 0.4327 | 1.0000 | 0.6400 | 1.0000 | 0.4494 | 0.3255 |
| NSZ-FEP | | N = 45 |  |  |  |  |  |  |  |
|  | Baseline - Week 6 | | 0.9993 | 0.9975 | 0.0893 | 0.9925 | 0.3633 | 0.9578 | 0.9121 |
|  | Baseline - Week 16 | | 0.1533 | 0.4226 | 0.4663 | 0.2646 | 0.9997 | 0.8824 | 0.1822 |
|  | Week 6 - Week 16 | | 0.0999 | 0.1380 | 0.4817 | 0.0520 | 0.5584 | 0.0848 | **0.0110** |
| SZ-FEP, schizophrenia first episode psychosis patients; NSZ-FEP, non-schizophrenia first episode psychosis patients.  Covariates included age and intracranial volume. p value corrected for multiple comparisons using Tukey’s HSD. | | | | | | | | | |

| **Table S16: Subgroup Mixed Model F-test results for Bilateral Subfield and Bilateral Hippocampus Volumes**  **(N: SZ-FEP = 48 NSZ-FEP = 45)** | | | | | | | | | | |
| --- | --- | --- | --- | --- | --- | --- | --- | --- | --- | --- |
|  | CA1 | | CA3 | | CA4 | | Subiculum | | Presubiculum | |
| Fixed Effects | *F* (df1, df2) | *p* | *F* (df1, df2) | *p* | *F* (df1, df2) | *p* | *F* (df1, df2) | *p* | *F* (df1, df2) | *p* |
| Age | 1.62 (1, 80.3) | 0.2067 | 1.27 (1, 90.1) | 0.2620 | 3.06 (1, 84.8) | 0.0837 | 1.24 (1, 86.7) | 0.2682 | 0.083 (1, 90.9) | 0.7743 |
| ICV | 15.46 (1, 185.7) | **0.0001** | 20.64 (1, 161.3) | **<.0001** | 22.40 (1, 147.6) | **<.0001** | 30.27 (1, 139.3) | **<.0001** | 55.49 (1, 137.7) | **<.0001** |
| **Time** | 8.34 (2, 121.9) | **0.0004** | 5.95 (2, 133.3) | 0.0033 | 6.61 (2, 128.4) | **0.0018** | 3.48 (2, 131.0) | **0.0337** | 5.12 (2, 135.7) | **0.0072** |
| **Group** | 0.91 (1, 80.2) | 0.3424 | 0.01 (1, 90.9) | 0.9358 | 0.05 (1, 85.8) | 0.8188 | 4.78 (1, 88.1) | **0.0314** | 3.86 (1, 92.7) | 0.0524 |
| **Group X Time** | 1.16 (2, 119.7) | 0.3163 | 0.77 (2, 131.3) | 0.4642 | 3.01 (2, 126.5) | 0.0529 | 1.59 (2, 129.3) | 0.2087 | 0.70 (2, 134.1) | 0.4975 |

| **Table S16: Continued** | | | | | | | | |
| --- | --- | --- | --- | --- | --- | --- | --- | --- |
|  | Molecular Layer | | GC/ML/DG | | Hippocampal Tail | | Whole Hippocampus | |
| Fixed Effects | *F* (df1, df2) | *p* | *F* (df1, df2) | *p* | *F* (df1, df2) | *p* | *F* (df1, df2) | *p* |
| Age | <0.01 (1,86.8) | 0.9514 | 3.24 (1, 84.8) | 0.0753 | 1.03 (1, 91.7) | 0.3138 | 2.02 (1, 84.1) | 0.1592 |
| ICV | 51.76 (1, 116.3) | **<.0001** | 23.99 (1, 144.0) | **<.0001** | 17.82 (1, 163.2) | **<.0001** | 32.91 (1, 182.0) | **<.0001** |
| **Time** | 1.95 (2, 133.8) | 0.1465 | 6.28 (2, 128.6) | **0.0025** | 2.07 (2, 134.8) | 0.1282 | 8.04 (2, 126.1) | **0.0005** |
| **Group** | 4.58 (1, 89.9) | **0.0351** | 0.05 (1, 85.9) | 0.8197 | 1.64 (1, 92.5) | 0.2032 | 1.59 (1, 84.1) | 0.2102 |
| **Group x Time** | 1.43 (2, 132.5) | 0.2431 | 3.35 (2, 126.8) | **0.0381** | 0.34 (2, 132.9) | 0.7130 | 3.04 (2, 123.9) | 0.0516 |
| ICV, intracranial volume. | | | | | | | | |

| **Table S18: Subgroup Mixed Model F-test results for Hippocampal Glutamate (N: SZ-FEP = 48 NSZ-FEP = 45)** | | |
| --- | --- | --- |
| Fixed Effects | *F* (df1, df2) | *p* |
| Sex | 13.71 (1, 186.0) | **0.0003** |
| PPD | 0.74 (1, 186.0) | 0.3906 |
| FWHM | 18.44 (2, 186.0) | **<.0001** |
| **Time** | 1.35 (1, 186.0) | 0.2624 |
| **Group** | 0.44 (2, 186.0) | 0.5094 |
| **Group x Time** | 0.49 (1, 186.0) | 0.6588 |
| PPD, packs per day; FWHM, full-width at half maximum. | | |

| **Table S19: Lateralized Hippocampal Subfield Volume Differences at Baseline in FEP** | | | | | |
| --- | --- | --- | --- | --- | --- |
|  | Volume MM^3^ | |  |  |  |
| Subfields | Left Hemisphere | Right Hemisphere | *t* | df | *p* |
| CA1 | 636.68 (85.73) | 667.71 (94.19) | 2.337 | 182 | **0.021** |
| CA3 | 213.45 (31.30) | 229.78 (35.21) | 3.324 | 182 | **0.001** |
| CA4 | 243.40 (31.43) | 251.61 (31.70) | 1.765 | 182 | 0.079 |
| subiculum | 415.99 (49.00) | 412.36 (43.69) | 0.530 | 182 | 0.597 |
| presubiculum | 283.59 (37.24) | 271.80 (32.72) | 2.281 | 182 | **0.024** |
| molecular layer | 502.67 (63.98) | 514.27 (68.27) | 1.189 | 182 | 0.236 |
| CG/ML/DG | 293.68 (37.76) | 301.77 (38.93) | 1.430 | 182 | 0.154 |
| Hippocampal tail | 553.46 (75.50) | 560.37 (72.64) | 0.633 | 182 | 0.528 |
| Total Hippocampus | 3350.85 (378.48) | 3409.57 (381.66) | 1.048 | 182 | 0.296 |

| **Table S20: Lateralized Anterior and Posterior Hippocampal Volume Differences at Baseline in FEP** | | | | | |
| --- | --- | --- | --- | --- | --- |
|  | Volume MM^3^ | |  |  |  |
|  | Left Hemisphere | Right Hemisphere | *t* | df | *p* |
| Posterior | 1205.00 (127.49) | 1166.85 (123.74) | 2.108 | 181 | **0.036** |
| Anterior | 1779.43 (249.03) | 1682.34 (217.35) | 2.810 | 181 | **0.005** |

| **Table S21: Week 16 Group Comparison of Match Demographics** | | | | |
| --- | --- | --- | --- | --- |
|  | FEP (n=70) | HC (n=57) | t/χ2 | p |
| Sex (%male) | 64.29 | 57.89 | 0.542 | 0.462 |
| Age | 23.81 (6.08) | 24.39 (5.98) | 0.531 | 0.596 |
| Parental SES*^a^* | 5.10 (4.69) | 4.63 (4.01) | 18.566 | 0.292 |
| *^a^*  SES determined from Diagnostic Interview for Genetic Studies (1–18 scale); lower numerical value correspond to higher socioeconomic status; 2 FEP lacked SES scores, N=68. | | | | |


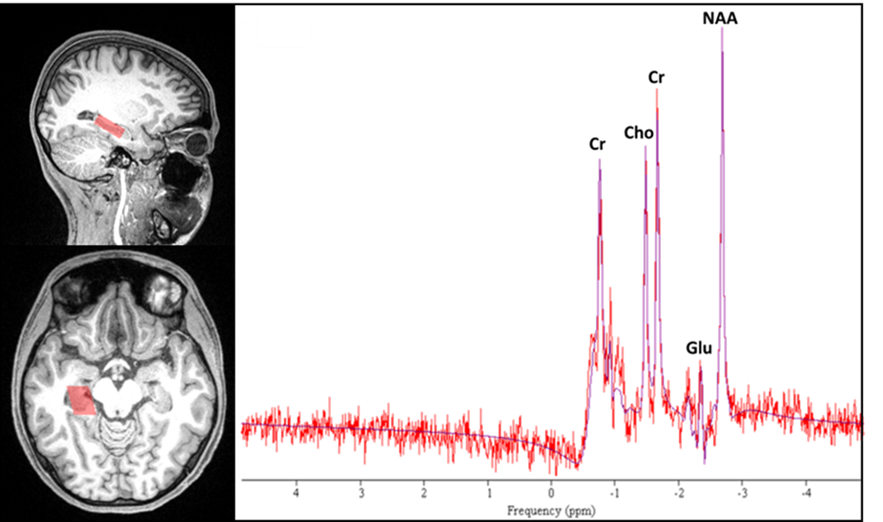


**Figure S1. *Hippocampal Spectroscopy Voxel and QUEST Model:***

Left image shows magnetic resonance spectroscopy single voxel location in the left hippocampus. The right image shows a single subject spectrum where the red line represents a collected spectra sample, and the purple line indicates the model fit obtained using the QUEST algorithm in jMRUI. Cr, creatine; Cho, choline; Glu,
